# Evaluation of Human Amniotic Membrane as a Wound Dressing for Split-Thickness Skin-Graft Donor Sites

**DOI:** 10.1155/2014/572183

**Published:** 2014-06-09

**Authors:** Denys J. Loeffelbein, Nils H. Rohleder, Matthias Eddicks, Claudia M. Baumann, Mechthild Stoeckelhuber, Klaus-D. Wolff, Enken Drecoll, Lars Steinstraesser, Simone Hennerbichler, Marco R. Kesting

**Affiliations:** ^1^Department of Oral and Maxillofacial Surgery, Munich University of Technology, Ismaninger Straße 22, 81675 Munich, Germany; ^2^Clinic for Swine, Faculty of Veterinary Medicine, Ludwig-Maximilians-University of Munich, Sonnenstraße 16/A106, 85764 Oberschleissheim, Germany; ^3^Department of Pathology, Munich University of Technology, Ismaninger Straße 22, 81675 Munich, Germany; ^4^Department of Plastic, Reconstructive and Aesthetic Surgery, European Medical School at the Carl von Ossietzky University of Oldenburg, Evangelisches Krankenhaus, Steinweg 13–15, 26122 Oldenburg, Germany; ^5^Red Cross Blood Transfusion Service of Upper Austria/Austrian Cluster for Tissue Regeneration, Krankenhausstrasse 7, 4017 Linz, Austria

## Abstract

Human amniotic membrane (HAM) has been used as a biomaterial in various surgical procedures and exceeds some qualities of common materials. We evaluated HAM as wound dressing for split-thickness skin-graft (STSG) donor sites in a swine model (Part A) and a clinical trial (Part B). Part A: STSG donor sites in 4 piglets were treated with HAM or a clinically used conventional polyurethane (PU) foil (*n* = 8 each). Biopsies were taken on days 5, 7, 10, 20, 40, and 60 and investigated immunohistochemically for alpha-smooth muscle actin (*α*SMA: wound contraction marker), von Willebrand factor (vWF: angiogenesis), Ki-67 (cell proliferation), and laminin (basement membrane integrity). Part B: STSG donor sites in 45 adult patients (16 female/29 male) were treated with HAM covered by PU foam, solely by PU foam, or PU foil/paraffin gauze (*n* = 15 each). Part A revealed no difference in the rate of wound closure between groups. HAM showed improved esthetic results and inhibitory effects on cicatrization. Angioneogenesis was reduced, and basement membrane formation was accelerated in HAM group. Part B: no difference in re-epithelialization/infection rate was found. HAM caused less ichor exudation and less pruritus. HAM has no relevant advantage over conventional dressings but might be a cost-effective alternative.

## 1. Introduction

Human amniotic membrane (HAM) is the inner layer of the fetal membranes (the outer layer being formed by the chorion) and has been investigated as an alternative biomaterial for various purposes in reconstructive surgery and wound-healing research since its initial description as a transplantable material by Davis in 1910 [1]. In the 20th century, it offered new perspectives, for example, in the treatment of burn wounds, as shown in a 1977 clinical study in which it was used as a dressing for second and third degree burns in children, exhibiting superior qualities when compared with conventional dressings [2]. In another exemplary study in 1982, amniotic membranes were used for the coverage of facial dermabrasions in thirty-three patients. The results “were excellent” and revealed “advantages of amniotic membranes over the other employed dressing techniques” [3]. However, interest in HAM research and clinical investigations diminished as a consequence of the emerging awareness of AIDS and the consequent fear of virus transmission in the 1980s. It was not before the end of the 1990s that new methods for the processing and long-term storage (cryopreservation) of HAM were established, and its use in wound care and reconstructive surgery became a target of scientific interest again [4]. For example, 2308 HAM transplantations for ophthalmologic reconstructions were carried out in Germany in 2008 [5]. Transplantation to the eye seems to be possible because of the immune-privileged properties of HAM [6]. This feature might also be an explanation for the lack of adverse reactions following HAM transplantations noted in other studies [2]. Amniotic membrane has been demonstrated to function as a tissue engineering scaffold material, for example, for corneal epithelium reconstruction by means of transplantation of epithelial cells on a lyophilized amniotic membrane (LAM) [7]. Interestingly, HAM further seems to release factors with antioncogenic potential, for example, for the inhibition of prostate cancer cell growth [8]. The combination of HAM (as a feeder layer) with other antioncogenic agents might result in synergetic effects and possibly in innovative potent materials for cancer treatment. Horch et al. have recently stated that the “synthesis of tissue engineering with innovative methods of molecular biology and stem-cell technology may help investigate and potentially modulate principal phenomena of tumour growth and spreading, as well as tumour-related angiogenesis” [9].

**Figure 1:**
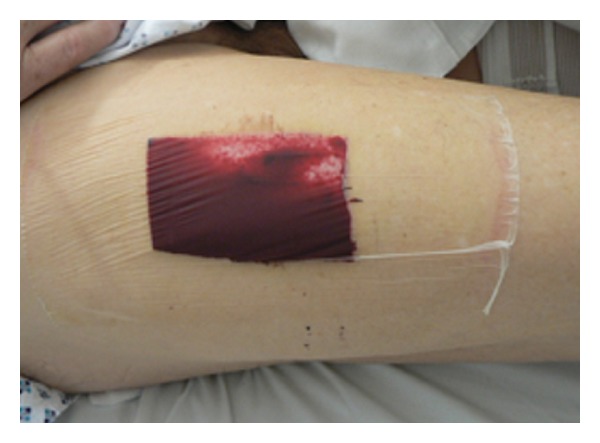
Wound dressing of a split-thickness skin-graft donor site defect (covered with PU foil) on the anterolateral thigh on the third postoperative day. Note the accumulation of wound exudate, which disturbs the clinical evaluation of reepithelialization. In addition, the risk of uncontrolled leakage is high, as is patient discomfort.

Recently, we have investigated the suitability of HAM as part of a wound dressing for full-thickness skin-graft donor sites in a porcine model and obtained promising results [10]. However, the usefulness of HAM as a dressing biomaterial for split-thickness skin-graft (STSG) donor sites has not been investigated yet. Commonly used dressing techniques such as polyurethane (PU) film or foam dressings are beset with disadvantages, such as the accumulation of blood and wound secretions that make visual monitoring of the wound-healing process difficult and imply the risk of uncontrollable leakage and infection (Figure 1). They are also expensive materials that cannot be afforded in all clinics, for example, in developing countries. The purpose of this study has been to evaluate the usefulness of HAM as an alternative biomaterial for STSG-donor site coverage and to compare the results with the commonly used PU foil and PU foam wound dressings in order to test the null-hypothesis that HAM exhibits superior qualities as a wound dressing when compared with PU foil/foam dressings. This hypothesis is based on the positive results obtained in previous clinical studies that demonstrated the qualities of HAM in the treatment of burn wounds and in ophthalmologic surgery and for the treatment of full-thickness skin-graft donor sites.

## 2. Materials and Methods

### 2.1. Part A

Animal experiments were approved by the local committee of animal welfare and by the local government (AZ: 9.93.2.10.32.07.152, LANUV Recklinghausen). Four six-month-old male Berlin minipigs (FA. Schlesier, Großerkmannsdorf, Germany) with initial body weights of 24–31 kg were caged according to international principles of laboratory animal care; water and food were available ad libitum.

Human placentas for the harvesting of HAM for use in the animal experiment were obtained and processed as previously described [10]. General anesthesia, intubation, and perioperative management of the animals were carried out according to previous protocols [11]. Until use, each HAM sheet used in the animal experiment was kept moist by storage in a sterile tube filled with a standard cryopreservation medium containing dimethyl sulfoxide (DMSO) as described earlier [10]. All HAM sheets for the animal experiment were immediately transferred to the laboratory on the same facility for processing (e.g., rinsing with sterile solution for removal of blood, selection of avascular areas, cutting into square pieces, and storage in DMSO cryopreservation medium) and snap-frozen. Hence, growth factors within the membranes were preserved.

**Figure 2:**
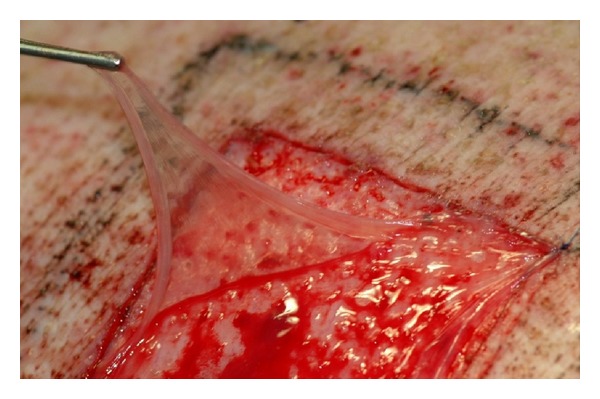
Application of human amniotic membrane to a porcine split-thickness skin-graft donor site defect.

Four square split-thickness skin-graft donor site defects (5 × 5 cm) were created on the back of each of the animals (sixteen wounds in total) with a dermatome (Nouvag GmbH, Konstanz, Germany) to a depth of 0.2 mm. Two of these four defects were randomly dressed with cryopreserved HAM (Figure 2), which were thawed 30 minutes before use in a 28°C water bath. Histological analysis of HAM revealed stromal tissue (connected to the chorion) on the inner side and an epithelial layer of cuboidal cells on the outer side. Both sides are divided by a basement membrane. In the present study, the smooth epithelial layer was identified by its glossy surface. The epithelial side of the HAM was subsequently placed onto the STSG-donor site defect surface. The two remaining defects were covered with a PU foil (3M Tegaderm Film, 3 M, St. Paul, Minnesota, USA) as a control. Thus, a total of eight defects were treated with HAM and a total of eight defects with PU foil. After application to a STSG-donor site, HAM was kept moist with an occlusive wound dressing. For stabilization and protection of the wound dressings against dirt, an additional cotton bandage (Rolta 10 cm × 3 m, Henry Schein Vet GmbH, Hamburg, Germany) and an elastic tape (CoFlex 10 cm × 4.5 m, Henry Schein Vet GmbH, Hamburg, Germany) were applied. The elastic tape was finally fixed with adhesive tape so that the pigs were not able to rip off the dressing. The bandages were renewed at least after every second to third day according to requirements. Animals were sacrificed 60 days after the surgical procedures by intravenous administration of T61 (Bayer, Leverkusen, Germany), at 1 mL/5 kg body weight.

#### 2.1.1. Evaluation of the Wound-Healing Process

The wound-healing process was evaluated on postoperative days 5, 7, 10, 20, 40, and 60 by photodocumentation with a digital camera (DMC-FZ4, Panasonic, Matsushita Electric Industrial Co., Ltd. Oska, Japan) and standardized with 6 mm tissue punch biopsies (PFM AG, Carlsbad) on the respective days. Samples were investigated histologically by hematoxylin/eosin (HE) staining for general tissue architecture and thickness of the epithelial layer (as an indicator of cicatrization/hypertrophy) and immunohistochemical staining for alpha-smooth muscle actin (*α*SMA, which is a marker for cicatrization), von Willebrand factor (vWF, in order to determine angiogenesis by the visualization of vessel walls), Ki-67 (as an indicator of proliferating cells), and laminin (as a component of the basement membrane, representing its integrity).

#### 2.1.2. Histological and Immunohistochemical Analysis

Samples were stored in formalin (3.7%) for 24 hours and embedded in paraffin, following which 4 mm thick sections were cut with a Hyrax M 55 microtome (Zeiss, Jena, Germany) and deparaffinized. Some of the sections were stained with hematoxylin/eosin (HE) in an automated staining system (Micro HMS 740 Robot-Stainer, Thermo Fisher Scientific, Waltham, USA) and analyzed with an AxioCam HRC microscope (Zeiss) to visualize and characterize the tissue architecture and epithelial thickness. The thickness of the epithelial layer was determined on days 7 and 60 by 48 measurements per biopsy at intervals of 0.05−1 mm of the vertical distance from the epithelial surface to the basement membrane by means of “Leica Q Win” software (Leica Microsystems, Wetzlar, Germany). The former time point represented the moment of complete reepithelialization of all wounds, with the latter corresponding to complete scar formation.

The remaining sections were immunohistochemically stained with primary antibodies against *α*SMA (1 : 80, mouse monoclonal to alpha-smooth muscle actin, Abcam, Cambridge, UK), vWF (1 : 200, polyclonal rabbit anti-human von Willebrand factor, Dako, Glostrup, Denmark), Ki-67 (1 : 50, monoclonal mouse anti-human Ki67-antigen, Dako), and laminin (1 : 50, polyclonal rabbit antilaminin, Dako) by using the Vectastain ABC Kit and a biotinylated secondary antibody (1 : 200; Vector Laboratories, Burlingame, CA, USA). The slides were incubated with peroxidase-conjugated streptavidin (Vector). Diaminobenzidine was used as a chromogen. The sections were counterstained with Mayer's hematoxylin (Bio Optica, Milan, Italy). Negative controls without the primary antibodies were treated identically. All staining was carried out in duplicate. The sections were viewed, and images were captured with a Leitz Aristoplan microscope (Leica, Wetzlar, Germany). Staining was scored independently by two investigators as follows: in order to determine the cellular basis for wound contractions and scarring, the number of *α*-actin positive myofibroblasts, which had been stained with the *α*SMA antibody, was counted within five high-power fields (HPF), 10 sections being evaluated in each group on each day.

Neovascularization was analyzed at the strongest phase of proliferation of wound-healing on days 7 and 10 by means of vWF staining. vWF is located in vessel walls and therefore makes the determination of the number of vessels possible.

The proliferative activity of the epithelium was determined by the calculation of the proliferation index, which is the quotient of the number of proliferating cells (stained by the Ki-67 antibody) and the total number of basal cells. Five HPF per biopsy were analyzed in each group on days 7 and 60 to map the moment of highest proliferation in the early phase of wound-healing and the proliferation status in scar tissue.

The integrity of the newly produced basement membrane was determined by the staining of laminin, one of its integral components. The presence of a basement membrane was assessed in 10 sections of each biopsy as being either “not visible” (−), “partly observable” (±), or “complete” (+).

### 2.2. Part B

**Figure 3:**
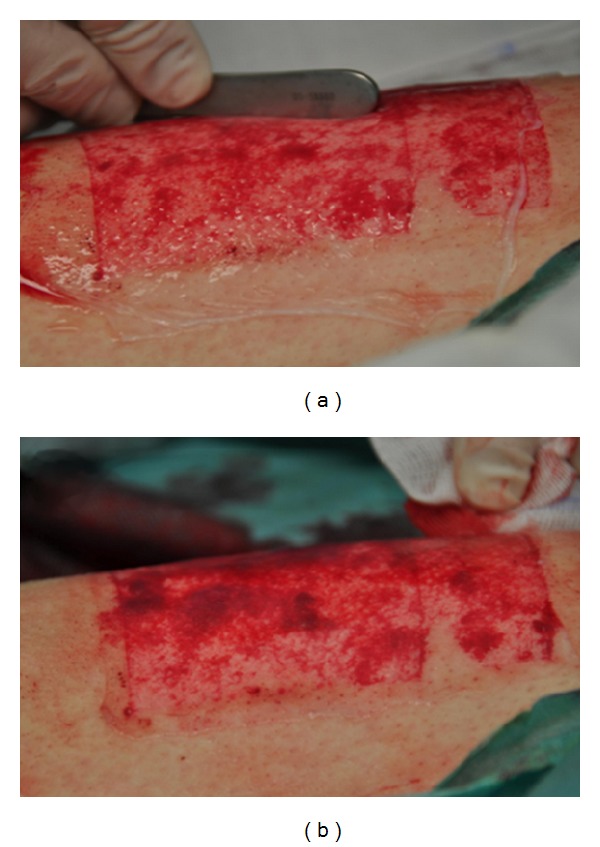
Application of allogenic human amniotic membrane on a human split-thickness skin-graft donor site defect on the lateral thigh. (a) Application is conducted in a way such that no air enclosures occur under the membrane. (b) Situation after trimming of the membrane with an overlapping zone of ~3 cm on the surrounding skin and fixation with fibrin glue.

The study protocol for the use of HAM was approved by the District Council and the local ethics committee (no. 3071/10; 1 February 2011). Operations were carried out in accordance with the Declaration of Helsinki. All patients gave written informed consent. In the context of reconstructive procedures, a standardized STSG of 0.4 mm (0.016 inch) thickness was harvested from the anterolateral thigh with a dermatome (Acculan 3Ti Dermatom, FA. Aesculap AG, Tuttlingen, Germany) from 45 patients. All patients had similar characteristics regarding their morbidity (neck dissection, resection of an intraoral tumor/lesion such as oral squamous cell carcinoma, defect reconstruction with a microsurgically anastomosed free flap transplant such as a radial forearm free flap, and closure of the donor site with a STSG from the thigh). The STSG-donor sites of the study group (*n* = 15) were covered with allogenic HAM (group A), with at least 3 mm overlapping and with the chorion site of HAM toward the wound ground. Allogenic HAM for the clinical study was fabricated in cooperation with the Red Cross Blood Transfusion Service of Upper Austria, Austrian Cluster for Tissue Regeneration, Linz, Austria, as a certified medicinal product with growth factor preservation conditions as previously described [12, 13]. Fibrin glue (Tissucol, Baxter, Vienna, Austria) was used on the wounds in a spraying technique to avoid shearing off through manipulation before the membranes were applied (Figure 3(a)). To avoid irritation by clothes after adaptation of the membrane to the wound (Figure 3(b)), HAM was covered by a PU foam (Mepilex, Mölnlycke Health Care, Erkrath, Germany). This procedure ensured sufficient stability of the HAM-dressing. In the first control group (*n* = 15), the wounds were covered directly and solely by a PU foam (Mepilex) (group B). In the second control group (*n* = 15), PU foil (3M Tegaderm Film, 3 M, St. Paul, Minnesota, USA) and consecutive paraffin gauze (Jelonet, Smith & Nephew GmbH, Marl, Germany) (group C) served as a cover of the STSG-donor site.

#### 2.2.1. Evaluation of the Wound-Healing Process

The clinical course was photo-documented on postoperative days 1, 3, 5, 7, 10, 12, 14, and 75 with a digital camera (DMC-FZ4, Panasonic, Matsushita Electric Industrial Co., Ltd. Osaka, Japan) and by a data survey questionnaire. The following clinical parameters were evaluated: exudation/dryness degree, number of dressing changes, pain sensation through dressing changes, pruritus, and dressing comfort. Quantitative assessment criteria were evaluated by two trained examiners on the basis of the questionnaire as follows: exudation of ichor: 0 = dry, 1 = visible exudate, 2 = moist visible exudate, 3 = wet exudate visible, and 4 = dripping exudate visible; amount of dressing changes: overall needed dressing changes; pain sensation: visual analog pain scale (0 = no pain to 10 = unbearable pain); pruritus: 0 = no itching, 1 = light, 2 = moderate, 3 = strong, and 4 = very strong. Subjective comfort: 0 = excellent, 1 = good, 2 = satisfactory, 3 = uncomfortable, and 4 = inacceptable.

#### 2.2.2. Statistical Analysis

IBM SPSS Statistics 19.0 software (SPSS, Inc., Chicago, IL, USA) was used for statistical calculations. Differences between the groups in epithelial thickness, the number of *α*-actin-positive myofibroblasts (*α*SMA staining), vessels (VWF staining), and the number of proliferating cells (Ki-67 staining) were analyzed by Student's* t*-test. The means of every clinical parameter of all patients over all postoperative controls were compared with reference to the used dressing material. Data were analyzed as unrelated measurements by the Mann Whitney* U*-Test. All *P*  values are given as being two-tailed and are subject to a local significance level of 5%.

## 3. Results

### 3.1. Part A

#### 3.1.1. Clinical Course and Histological Overview

**Figure 4:**
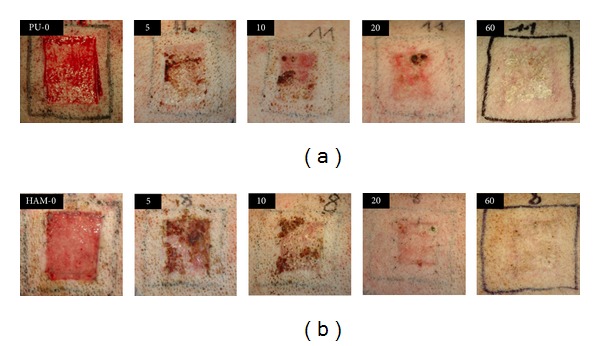
Clinical course of split-thickness skin-graft donor site wounds from days 0 to 60. Upper row shows a representative PU-treated wound, whereas the lower row shows a representative HAM-treated wound. Note the lack of significant difference in the wound-healing progress.

No difference in the speed of macroscopic wound-healing was evident between the groups (Figure 4). During postoperative progress, the two groups showed no differences with regard to bleeding (slight hemorrhages were observed in both groups during the first few days after operation), inflammation, infection, or chronological sequence of wound-healing. Macroscopic wound contraction measurements revealed no contraction in either group. In the HAM group, seven wounds (87.5%) showed a final skin-like color on day 60 (the remaining wound exhibited a pink color), whereas four wounds (50%) exhibited a skin-like color in the PU group (of the remaining wounds, 3 were pink, 1 was white).

Histological evaluation of the wound-healing process by means of HE staining revealed no relevant differences between the groups; cell infiltration, reepithelialization, and maturation of the epithelial layers occurred at almost the same time in the HAM and PU groups.

#### 3.1.2. Epithelial Thickness

Measurement of the epithelial thickness revealed a significantly broader epithelium in the HAM group on day 7 (*P* < 0.001). By contrast, the epithelium in the HAM group was significantly thinner than in the PU group on day 60 (*P* < 0.001). The epithelium in the HAM group was thinner on day 60 than on day 7 (*P* < 0.001); epithelial thickness did not change in the PU group between days 7 and 60 (Table 1).

#### 3.1.3. Immunohistochemical Staining of *α*SMA: Wound Contraction

No *α*SMA-positive cells were identified in any of the specimens of any group because of nonexistent myofibroblasts in the specimens. Accordingly, no statistical comparison was applicable.

#### 3.1.4. Immunohistochemical Staining of vWF: Neovascularization

**Figure 5:**
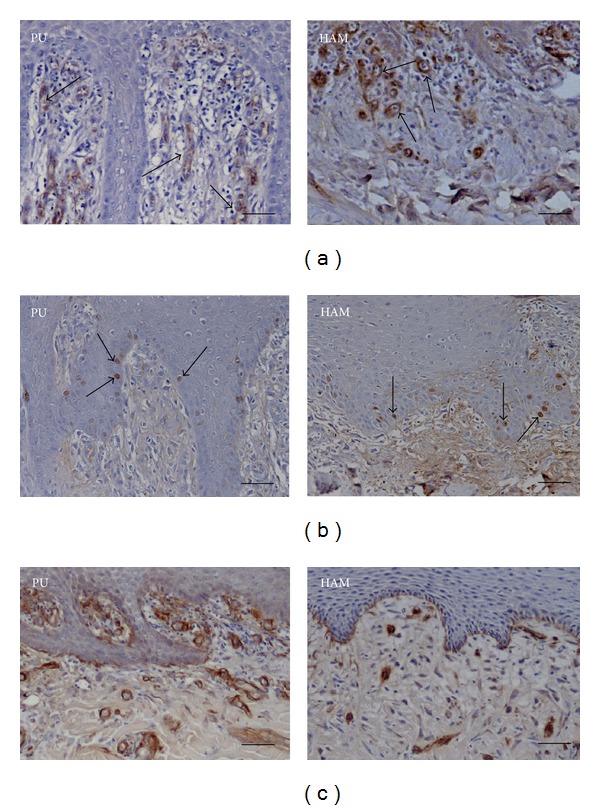
(a) Marking of vascular walls with immunohistochemistry for von Willebrand factor. Polyurethane-dressed wound on the left and human-amniotic-membrane-dressed wound on the right (300-fold magnification, scale bar: 50 *μ*m) on the tenth postoperative day. The arrows point exemplarily to vessel walls that are seen as circular structures with a brownish color because of staining with primary and secondary antibodies. (b) Marking of proliferating cells by immunohistochemistry for Ki-67. Polyurethane-dressed wound on the left and human-amniotic-membrane-dressed wound on the right (300-fold magnification, scale bar: 50 *μ*m) on the seventh postoperative day. The arrows point exemplarily to proliferating cells that have a brownish color because of the staining with primary and secondary antibodies. (c) Marking of a basement membrane by immunohistochemistry for laminin. Polyurethane-dressed wound on the left and human-amniotic-membrane-dressed wound on the right (300-fold magnification, scale bar: 50 *μ*m) on the tenth postoperative day. Note the complete integrity of the basement membrane in the HAM example.

The number of vessels was significantly higher in the PU group than in the HAM group on both day 7 and day 10 (*P* < 0.001; Figure 5(a)). The number of vessels was higher on day 10 than on day 7 within both groups (significant only in the PU group; *P* < 0.001). All numbers are detailed in Table 2.

#### 3.1.5. Immunohistochemical Staining of Ki-67: Proliferation

No difference in proliferation indices was evident between the groups on postoperative days 7 and 60 (Figure 5(b)). The proliferation index was significantly higher on day 60 within both groups (Table 3).

#### 3.1.6. Immunohistochemical Staining of Laminin: Formation of Basement Membrane

Samples of HAM-treated wounds exhibited a complete basement membrane at day 10 (Figure 5(c)). A comparable integrity of the basement membrane was not observable until day 20 in the PU group (Table 4).

### 3.2. Part B

#### 3.2.1. Clinical Course: Macroscopic Evaluation

The sizes of the donor sites were between 18 and 32 cm^2^ and did not differ significantly between the three groups. Wound-healing was uneventful in 41 of 45 patients. In 4 patients (1 × HAM/PU, 1 × PU, and 2 × PU/Gauze), infection of the wound was suspected before postoperative day 12. Treatment was initiated with topical application of polyhexanide-gel twice a day.

**Figure 6:**
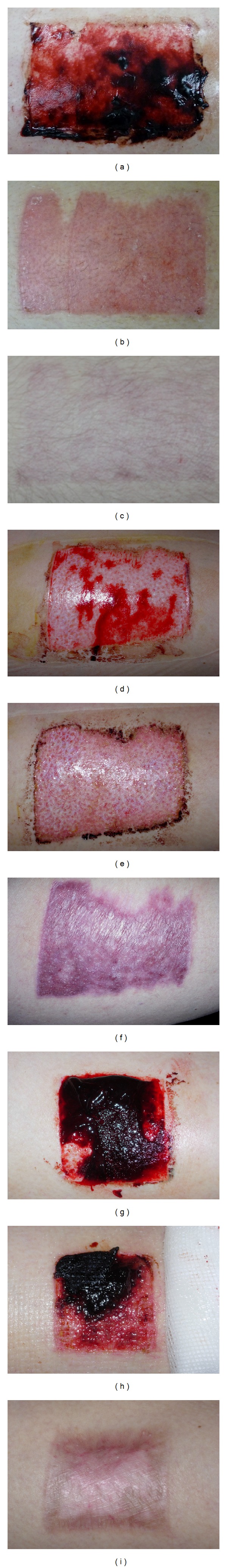
Clinical course of human split-thickness wounds covered with HAM and PU foam (a–c), PU foam alone (d–f), and PU foil/gauze (g–i) on days 1, 12, and 75 (each row from left to right).

In the study group, 93.3% of the wounds were completely epithelialized on postoperative day 12, whereas 86.7% were epithelialized in both control groups. This revealed no significant difference. The macroscopic clinical course of wound-healing in all three groups is displayed in Figure 6 (donor site wounds covered with HAM and PU foam (a–c), PU foam alone (d–f), and PU foil/gauze (g–i) on days 1, 12, and 75; each row from left to right).

#### 3.2.2. Wound Exudation

**Figure 7:**
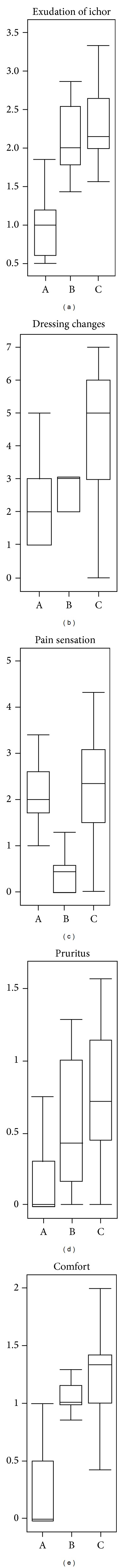
Box plot diagrams illustrate the results of a comparison between group A (wounds treated with HAM, *n* = 15), group B (first control group of *n* = 15 wounds, covered directly and solely by polyurethane foam), and group C (second control group of *n* = 15 wounds in which polyurethane foil and consecutive paraffin gauze served as a cover of the STSG-donor site). (a) shows the exudation of ichor on a scale from 0 to 4 (0 = dry; 1 = visible exudate; 2 = moist visible exudate; 3 = wet exudate visible; 4 = dripping exudate visible). (b) shows the overall required amount of dressing changes during the first 14 postoperative days. (c) shows the relative pain sensation value (visual analog pain scale: 0 = no pain to 10 = unbearable pain) during dressing changes recorded by the patients. (d) shows the mean of pruritus development during the first 14 days of wound-healing (0 = no itching; 1 = light; 2 = moderate; 3 = strong; 4 = very strong). (e) shows evaluation of comfort as reported by the patients (0=excellent; 1 = well; 2 = satisfactory; 3 = uncomfortable; 4 = inacceptable).

Significantly less wound exudation was found in HAM-treated wounds (*n* = 15, group A) compared with the first control group of *n* = 15 wounds covered directly and solely by a PU foam (Mepilex; group B) and with the second control group (*n* = 15) in which PU foil (3M Tegaderm Film, 3 M, St. Paul, Minnesota, USA) and consecutive paraffin gauze (Jelonet, Smith & Nephew GmbH, Marl, Germany) (group C)served as a cover of the STSG-donor site (A versus B, A versus C; both *P* < 0.001). No differences were found regarding the dryness degree between both control groups (B versus C; *P* = 0.210; Figure 7(a)).

#### 3.2.3. Amount of Dressing Changes

Groups A and B required fewer dressing changes compared with group C (A versus C and B versus C; both *P* < 0.05). Compared with each other, no significance was evident between groups A and B (*P* = 0.332; Figure 7(b)).

#### 3.2.4. Pain Sensation

Pain sensation during dressing changes was significantly less in study group B compared with group A (B versus A and B versus C; both *P* < 0.001). No difference was evident between groups A and C (*P* = 0.368; Figure 7(c)).

#### 3.2.5. Pruritus

HAM-treated wounds (group A) showed the slightest pruritus of all groups during the initial phase of wound-healing (A versus B and A versus C; both *P* < 0.05). No difference was evident between groups B and C (*P* = 0.145; Figure 7(d)).

#### 3.2.6. Comfort

Regarding the comfort of the wound dressings as reported by the patients, the HAM-dressing exceeded the comfort of both control groups (A versus B and A versus C; both *P* < 0.001). Furthermore, the valuation of the comfort in group B was also significantly better evaluated than that in group C (*P* < 0.05; Figure 7(e)).

## 4. Discussion

STSG are widely applied in all fields of reconstructive surgery such as skin cancers, burns, and extensive wounding. Under normal conditions, the donor site heals by reepithelialization from the dermis (epithelium grows out from hair follicles) and from surrounding skin but requires dressings for the first two to three weeks. The ideal dressing for an STSG-donor site should promote the rate of reepithelialization, control the exudation to a physiological level, avoid leakages, and be comfortable for the patient with regard to pain and pruritus and the number of dressing changes. The postoperative course of wound-healing significantly depends on the degree of inflammation and infections. An anti-infective effect of HAM has been reported [14, 15]. This seems to be a result of the synthesis of anti-inflammatory proteins and of a reduction of the expression of transforming growth factor-b (TGF-b) and proinflammatory cytokines, such as interleukin-10 (IL-10) [14, 16]. Amnion cells synthesize peptides of the innate immunity system, such as *β*-defensins, elastase-inhibitors, elafin, lactoferrin, or IL-1-RA; these factors might be the effectors of the antimicrobial capacities of HAM [17, 18]. In the present study, split-thickness wounds treated with HAM showed almost no infections, which is in accordance with the above-mentioned reports demonstrating the anti-infective capacities of HAM. In addition to its endogenous factors, another reason responsible for the low rate of infections in defects covered with HAM might be its capacity of wound adherence [19].

Analysis of epithelial thickness allows an assessment of scar hypertrophies. Scars typically develop a few weeks after trauma and initially show erythroid colorization [20]. Within three to six months, the scar tissue proliferates and increases in diameter until a steady state is reached. Subsequently, the scar partially reduces its erythroid colorization and thickness over a period of about one year [21]. In the animal model used in this study, measurement of the epithelial thickness in the STSG wounds revealed a significantly higher epithelium in the PU group than in the HAM group on postoperative day 60, that is, a tendency for increased cicatrization. The reduction of cicatrization in the HAM group might be attributable to its anti-inflammatory capacities and its previously described accelerating effect on reepithelialization [4, 14, 16, 19] and to an inhibition of fibrosis [22]. With respect to the esthetic results in our investigation, HAM-treated defects in the animal model showed skin-colored epithelium in more cases (7/8 animals; 87.5%) than in the PU group (4/8 animals; 50%). A similar tendency was observed in the clinical part of this study after 72 days of observation. These findings are of potential clinical relevance. However, these results will have to be reevaluated/verified in a larger (multicentered) controlled clinical trial.

No contractions were observable in any group, either experimental or clinical. In the animal model, we performed a quantification of wound contraction by immunohistochemical staining of *α*SMA. The basis for this method is the finding that *α*SMA as an isoform of actin is located within the cytoskeleton of myofibroblasts and participates in cell motility. Myofibroblasts are located in healthy tissue and in pathologically altered tissues; a positive correlation of their number has been reported in various diseases, such as Dupuytren's contracture, and also with the degree of hypertrophy in scars [23, 24]. However, no *α*SMA-positive cells were identified within the epithelial layers of any biopsy in both groups. This is in agreement with the observation of no wound contractions by macroscopic evaluation. Accordingly, an inhibitory effect on wound contractures of HAM cannot be postulated based on the results of this investigation. Fraser et al. have reported reduced scar tissue formation and *α*SMA content (analyzed by *α*SMA staining) after the treatment of burn wounds in lambs with HAM (paraffin gauze was used as a control) [25]. With respect to the present study, this indicates differences between various animal models concerning the involvement of *α*SMA in wound regeneration, an aspect that should be considered in subsequent investigations.

Another issue that has not yet been sufficiently investigated is the influence that HAM exerts on angiogenesis, which is an important factor for the success of numerous surgical interventions and for wound-healing [26]. Irrespective of the particular pathology, a typical characteristic of chronic nonhealing wounds is an insufficient blood circulation as a consequence of the reduced formation of blood vessels. Hao et al. have reported the expression of thrombospondin-1, which is an antiangiogenetic protein, in all epithelial and in ~20% of the mesenchymal cells of HAM [14]. The authors have also demonstrated the expression of metalloprotease inhibitors, TIMP-1, -2, -3, and -4, which also exhibit a potent antiangiogenetic effect. By contrast, other studies in which HAM has been used for transplantation have revealed angiogenetic effects of HAM. For example, the authors of a study describing the use of HAM for the initial coverage of chronic leg ulcers before the application of autografts postulate a proangiogenetic capacity of HAM, because of the observed accelerated formation of granulating tissue [27, 28]. The experimental part of our study has shown a significantly lower number of vessels in the HAM group than in the PU group on postoperative day 5 and on postoperative day 7. This supports the above-mentioned studies postulating an antiangiogenetic effect of HAM. The reduced angiogenesis in the wounds might contribute to the more natural, skin-like color of the majority of the healed defects in the HAM group, whereas the denser vascularization in the wounds treated with polyurethane covers might be one reason for the pink/erythroid final wound colorization in these groups. However, further studies focused on this issue need to be conducted in order to evaluate more extensively angiogenesis in wounds treated with HAM.

The activation of keratinocytes was evaluated by Ki-67 staining in the animal part of the study in order to determine the speed of reepithelialization. Ki-67 interacts with a nuclear antigen synthesized in cells during the G1-, S-, M-, and G2-phases of the cell cycle and therefore is considered to be a marker for proliferating cells [29, 30]. During the early wound-healing process, the activation of keratinocytes plays a fundamental role in epithelial remodeling [31], with prolonged proliferation probably being associated with hypertrophic scarring [32]. HAM synthesizes numerous growth factors such as epithelial growth factor (EGF), keratinocyte growth factor (KGF), human growth factor (HGF), basic fibroblast growth factor (bFGF), and tissue growth factors (TGF-*α*, TGF-*β*-1, TGF-*β*-2, and TGF-*β*-3) and is assumed to accelerate reepithelialization and wound-healing by the activation of keratinocytes [19, 33, 34]. However, no differences in the rate of reepithelialization between the HAM and PU groups were seen in the experimental part of our study; both groups showed a clinically and histologically similar wound-healing process. Proliferation indices as a marker for the activation of keratinocytes did not differ between the groups on the investigated postoperative days. The clinical part of this study has revealed a nonsignificant difference in speed of reepithelialization in favor of HAM-treated wounds. Only a few comparable studies are available. Maral et al. have demonstrated an accelerated reepithelialization after the coverage of STSG defects in rats with autologous skin grafts, with the second-fastest reepithelialization being observed after coverage with HAM and the slowest healing in the untreated group [35].

Regular reepithelialization requires not only the closure of the wound surface but also the complete regeneration of the basement membrane, which plays a decisive role for the integrity and functionality of skin. The basement membrane is mainly composed of collagen type IV and laminin and is pivotal for coherence between the epithelial and dermal layers [36]. Andree et al. investigated the formation of the basement membrane during wound-healing of full-thickness skin-graft defects in a porcine model. The defects were covered with various epidermal transplants. The authors demonstrated a correlation between the transplant materials and the rate of recreation of the basement membrane [37]. In the animal model part of this study, we have observed an accelerated formation of the basement membrane in those wounds treated with HAM; all defects in this group show a complete basement membrane on postoperative day 10. By contrast, only incomplete fragments of the basement membrane are found in the PU group at this time. These findings might be explained on the basis of the above-mentioned release of growth factors by HAM.

The clinical trial further revealed that dressing changes were perceived as being more painful when wounds were treated with HAM-PU/gauze than when wounds were treated with PU foam without HAM. Significantly reduced wound exudation, less pruritus, and fewer dressing changes (with the highest subjective comfort) were observed in HAM-treated wounds. These results are in accordance with a study by Branski et al. who covered partial-thickness (second degree) burns in children with either HAM or topical antimicrobials; the patients in the amnion group needed significantly fewer dressing changes, but the rate of infections between the groups was not different [38].

We need to clarify whether HAM can be standardized for clinical use. The material can indeed be standardized by applying protocols provided by certified tissue banks. However, various culture or cryopreservation techniques are still under investigation [13, 39, 40]. For example, a quality check of each placenta for the HAM used in this study was carried out, and only avascular HAM areas were processed. HAM can further be standardized with respect to the selection of donors (e.g., exclusion of HIV-positive donors) and the testing of the material for infectious agents (e.g., agglutination tests for HIV (anti-HIV), HEP-C (anti-HCV), HEP-B (anti-HBc, HB-Ag), CMV (anti-CMV-IgG/-IgM), and TPHA- and ELISA-tests for syphilis and polymerase chain reaction tests for HIV, HCV, and HPV). As a consequence, we can assume that wound-healing should not be different between patients when standardized HAM is used for the treatment of similar defects, and the probability of disease transmissions should be similar to that of other allogeneic grafts such as blood cell concentrates.

Although HAM did not exceed the qualities of conventional materials to a relevant degree, it has shown almost equal characteristics. Previous studies have described HAM as a cost-effective treatment of burn wounds for developing countries [41, 42]. Accordingly, HAM might also be used as an economically reasonable alternative biomaterial for the treatment of STSG-donor sites in developing countries. HAM can be harvested and stored in any country fulfilling the following requirements: abdominal caesarean sections have to be carried out, and sterile conditions for the processing and storage of HAM must be available. To avoid ethical conflicts, institutional review board or ethics committee approval should be obtained, and local legal regulations should be met prior to the medical use of HAM. Being an allogeneic graft, similar to blood cell concentrates, no ethical concerns should be raised against the use of HAM in specific countries, as long as other allogeneic graft products are also used in clinical routine.

## 5. Conclusions

In view of the above-mentioned findings obtained in the animal model study and the clinical trial, treatment with HAM as a wound dressing for split-thickness wounds seems to result in improved esthetic results and in less hypertrophic scarring when compared with treatment with conventional PU-covered wounds during the first 75 days of wound-healing. Although no significant difference in the overall speed of reepithelialization is evident in this investigation, the accelerated reformation of the basement membrane might result in improved defensive capacities of the wound against microbial infections, since the basement membrane forms a line of resistance, even if the overlying epithelial layer is not complete. This should be investigated in further studies. The results of this combined experimental and prospective clinical trial reveal that HAM is a well-performing wound dressing for STSG-donor sites with statistically significant but clinically only minor (or even not relevant) advantages when compared with the commonly used PU dressings. However, HAM might be a cost-effective alternative wound dressing for STSG-donor sites in developing countries.

## Figures and Tables

**Table 1:** Epithelial thickness after treatment of split-thickness skin-graft donor sites with human amniotic membrane or polyurethane foil.

Wound dressing material	Mean value ± standard error of the mean (*μ*m)	
	The 7th postoperative day	The 60th postoperative day
Human amniotic membrane	653.3 ± 53.83	190.5 ± 12.24

Polyurethane foil	381.3 ± 23.45	324.8 ± 20.88

Control sample (untreated porcine skin)	168.0 ± 6.83	

**Table 2:** Number of vessels in porcine split-thickness skin-graft donor sites covered with either human amniotic membrane or polyurethane foil on postoperative days 7 and 10 (evaluated by immunohistochemical staining with von Willebrand factor antibody).

Wound dressing material	Mean value ± standard error of the mean	
	(number of vessels per high-power field)	
	The 7th postoperative day	The 10th postoperative day
Human amniotic membrane	0.90 ± 0.18	1.05 ± 0.14
Polyurethane foil	1.80 ± 0.16	4.70 ± 0.47

**Table 3:** Proliferation indices in samples of split-thickness skin-graft donor sites treated with human amniotic membrane or polyurethane foil on postoperative days 7 and 60 (evaluated by immunohistochemical staining with Ki-67 antibody).

Wound dressing material	Mean value ± standard error of the mean	
	of proliferation index	
	The 7th postoperative day	The 60th postoperative day
Human amniotic membrane	0.13 ± 0.0023	0.20 ± 0.0051
Polyurethane foil	0.15 ± 0.014	0.29 ± 0.0069

**Table 4:** Integrity of basement membrane in split-thickness skin-graft donor sites treated with human amniotic membrane or with polyurethane foil (evaluated by immunohistochemical staining with laminin antibody).

Postoperative day	Integrity of basement membrane in wounds	
	treated with	
	Human amniotic membrane	Polyurethane foil
5	±	±
7	±	±
10	+	±
20	+	+
40	+	+
60	+	+

Basement membrane (±) partly observable; (+) complete.
